# Assessing Stress and Shift Quality in Nursing Students: A Pre- and Post-Shift Survey Approach

**DOI:** 10.3390/healthcare13141741

**Published:** 2025-07-18

**Authors:** Haneen Ali, Yasin Fatemi

**Affiliations:** 1 Health Services Administration Program, Auburn University, Auburn, AL 36849, USA; 2 The Department of Mechanical and Industrial Engineering, Applied Science Private University, Amman 11937, Jordan; 3 Department of Industrial and Systems Engineering, Auburn University, Auburn, AL 36849, USA

**Keywords:** nursing students, single-shift quality, pre- and post-shift, survey instrument, stress perception

## Abstract

Background: Nursing students often experience heightened levels of stress during clinical training due to the dual demands of academic and clinical responsibilities. These stressors, compounded by environmental and organizational factors, can adversely affect students’ well-being, academic performance, and the quality of patient care they deliver. Aim: This study aimed to identify the key stressors influencing nursing students’ perceptions of single-shift quality (SSQ) during clinical training and to examine how well students can predict the quality of their shift based on pre-shift expectations. Methodology: A cross-sectional survey design was implemented, collecting pre- and post-shift data from 325 nursing students undergoing clinical training in Alabama. The survey measured 13 domains related to workload, environmental conditions, organizational interactions, coping strategies, and overall satisfaction. Paired t tests and linear regressions were used to assess changes in perception and identify key predictors of SSQ. Results: This study found significant discrepancies between students’ pre- and post-shift evaluations across multiple domains, including internal environment, organizational interaction with clinical faculty/preceptors, and coping strategies (*p* < 0.001). Students also accurately predicted stable factors such as patient characteristics and external environment. Pre-shift expectations did not significantly predict post-shift experiences. Post-shift perceptions revealed that stress-coping strategies and collegiality were the strongest predictors of shift quality. Conclusion: Students enter clinical shifts with optimistic expectations that often do not align with actual experiences, particularly regarding support and stress management. The SSQ framework offers a valuable tool for identifying gaps in clinical training and guiding interventions that foster resilience and better alignment between expectations and real-world practice.

## 1. Introduction

Nursing students experience greater stress than many of their undergraduate peers, with numerous studies identifying the need for early intervention and support mechanisms to mitigate its effects [1,2]. Clinical training, in particular, presents unique psychological and physical demands that significantly contribute to students’ stress levels. These demands stem from transitioning into unfamiliar clinical environments, interacting with new medical equipment, and a fear of making errors in high-stakes settings [1,2,3,4]. The cumulative impact of these stressors extends beyond individual discomfort, affecting students’ academic success, health behaviors, and even the quality of patient care they deliver [1,2,5]. Research has linked this stress to outcomes such as poor sleep, disordered eating, increased substance use, and compromised immune functioning, all of which threaten both personal and professional performance [3,4].

Compounding these challenges, the COVID-19 pandemic introduced additional, unprecedented stressors. Nursing students globally reported heightened levels of anxiety and depression, with surveys indicating that more than one-third experienced levels of stress so overwhelming that it interfered with their ability to concentrate [1,6]. The pandemic amplified the demands of clinical education and underscored existing weaknesses in how we measure and respond to student stress [3,4,7,8,9].

While several instruments exist to measure stress and safety culture among clinical staff—such as the AHRQ Hospital Survey of Patient Safety and the Safety Attitude Questionnaires (SAQs)—these tools are often designed for use with practicing nurses rather than students and typically assess organizational factors over longer periods [5,7,10]. Importantly, they rarely capture the more transient, shift-level stressors that can shape students’ immediate clinical perceptions and learning outcomes. In addition, they do not fully reflect the dynamic, person-specific variables, such as team composition and peer interaction, that change from one shift to the next and may disproportionately affect student experiences [5,7,11,12].

Recent work by Lu et al. (2013) on single-shift quality (SSQ) in neonatal nursing units suggests that individuals’ perception of their team and workload at the start of a shift can influence their appraisal of the overall shift experience [7]. However, their findings also show a mismatch between pre-shift expectations and post-shift evaluations, suggesting that even experienced nurses struggle to anticipate how contextual factors will unfold across a workday. Similar inconsistencies have been found in the literature, with limited insight into how these patterns manifest among students in training [5,7].

Given the absence of student-centered, real-time measurement tools, there remains a critical need to develop methods that better capture how stress unfolds within a single shift and how it affects nursing students’ perceived learning and well-being. This study addresses that gap by testing a cross-sectional survey instrument that evaluates daily clinical stressors through 13 domains: six explanatory domains for stressors, three physical/social domains for stress symptoms, and four domains for coping behaviors. These were derived from the existing literature and expert feedback.

The purpose of this study is to identify and explain daily stress factors experienced by nursing students during clinical shifts and to examine whether a relationship exists between perceived stressors and their expectations of experiencing adverse physical and social symptoms. Additionally, this study evaluates whether students can accurately predict their stress levels at the beginning of a shift and how their perceptions of stressors before and after the shift shape their overall evaluation of SSQ. By exploring these dynamics, we aim to provide educators and clinical supervisors with actionable insights to better support students in high-stress environments.

## 2. Methods

A cross-sectional survey was developed and distributed to nursing students during their clinical training in hospital settings across Alabama. Over a two-year period, a total of 325 students initially responded to the survey. However, 21 incomplete responses were excluded, resulting in a final sample of 304 participants included in the analysis. The participants were invited via email and word-of-mouth recruitment, which encouraged sharing with peers who met the inclusion criteria. The survey was administered online. Eligibility was limited to nursing students actively enrolled in clinical training programs within the state of Alabama.

The survey instrument included pre- and post-shift versions designed to assess nursing students’ perceptions of stressors and shift quality. The responses were gathered across six domains.

All data was analyzed using Python 3.11.9. Descriptive statistics were computed to summarize participant characteristics and response distributions. Paired t tests were conducted to evaluate statistically significant differences between pre- and post-shift responses. Pearson’s correlation analysis assessed associations among domain variables before and after the shift. To identify the key predictors of shift quality, multiple linear regression models were constructed using domain scores as independent variables and overall shift quality as the dependent variable, both before and after the shift.

### Description of the Survey Instrument

A survey tool was developed in two versions, one administered at the beginning of a clinical shift and the other at the end, to capture nursing students’ perceptions of stress and satisfaction during clinical training. Each version included 61 items across seven domains: demographics (5 items), workload (23 items), internal environmental factors (9 items), external environmental factors (3 items), organizational interaction with clinical training faculty or preceptors (7 items), stress-coping strategies (13 items), and overall satisfaction (1 item). All items were measured using a 5-point Likert scale.

The survey content was informed by a comprehensive review of the relevant literature and refined using expert input obtained through focus groups and elite interviews with nursing students and faculty. Feedback from academic and clinical stakeholders, including nursing faculty, public health administrators, and student representatives, was incorporated to enhance content validity. The instrument underwent pilot testing and was refined based on the responses and feedback collected

## 3. Domains Tested

### 3.1. Tasks

In this study, “tasks” refer to various dimensions of nurse workload, drawing from the frameworks of Carayon, Gurses, Karsh, and Holden [13,14,15]. We examined two key levels of workload: unit-level and job-level.

This domain assesses nursing students’ perceived workload during clinical shifts. It includes job-level workload [16] (task volume and time pressure), unit-level workload [17,18,19,20] (staffing ratios, handoff communication, and team coordination), and task-level [13,21] challenges (such as unfamiliar patients or missing supplies). These workload dimensions directly impact stress levels, task completion, and perceived shift quality.

### 3.2. Internal Environmental Factors

Internal environmental factors refer to the physical conditions within the clinical setting—such as lighting, noise, temperature, odors, and the physical layout of the unit. Distractions like excessive noise or poor cleanliness can hinder focus, contribute to fatigue, and negatively influence students’ clinical experience and performance [22,23,24].

### 3.3. Organizational Factors

This domain focuses on institutional structures that affect the clinical shift, including leadership visibility, communication with supervisors, teamwork, and interprofessional collaboration. Supportive environments that emphasize communication and leadership engagement enhance satisfaction and reduce stress, while disorganized or unsupportive structures can lead to confusion and anxiety [25,26,27].

### 3.4. External Environment

The external environment includes personal and contextual factors that influence students before a shift begins, such as fatigue, sleep quality, emotional state, and time of day. These factors shape readiness and resilience, affecting how students respond to clinical challenges during their shifts [15,28,29].

### 3.5. Stress Coping Strategies

This domain explores how students attempt to manage stress through various coping mechanisms. These may include avoidance behaviors, active problem-solving, or seeking emotional support from peers or professionals. The ability to use effective strategies during clinical shifts plays a vital role in emotional regulation and overall shift satisfaction [8,9,30,31,32].

### 3.6. Overall Satisfaction

Overall satisfaction is the final domain that captures nurses’ perceptions of their general satisfaction with their shifts.

Framework and definitions. This study uses the SSQ framework to explore how nursing students’ perceptions evolve from the beginning to the end of a clinical shift. The framework highlights how students form expectations based on prior experiences and how those expectations shift after real-time exposure to stressors, support, and workload. A key feature of the model is its cyclical nature: post-shift experiences inform future pre-shift expectations, which then shape ongoing perceptions of stress and satisfaction [7,33].

The following definitions clarify how each stage contributes to the SSQ process:

### 3.7. Definitions

Pre-shift expectations: initial perceptions based on anticipated shift tasks, responsibilities, and clinical environment.

Overall expectations: the importance students place on various domains (e.g., workload, faculty support) before the shift starts.

Post-shift expectations: actual events and challenges encountered during the clinical shift.

Overall satisfaction: post-shift reflection of how well the day aligned with initial expectations and goals.

Predictive ability: how accurately students’ pre-shift perceptions anticipated their actual experiences.

Change in context: the difference between what students expected and what they experienced, especially regarding stressors and support.

Change in SSQ expectations: adjustments in how students prioritize or view key domains based on recent clinical exposure.

Change in perceptions: long-term shifts in students’ mental models, influencing how future shifts are approached and interpreted.

**Figure 1:**
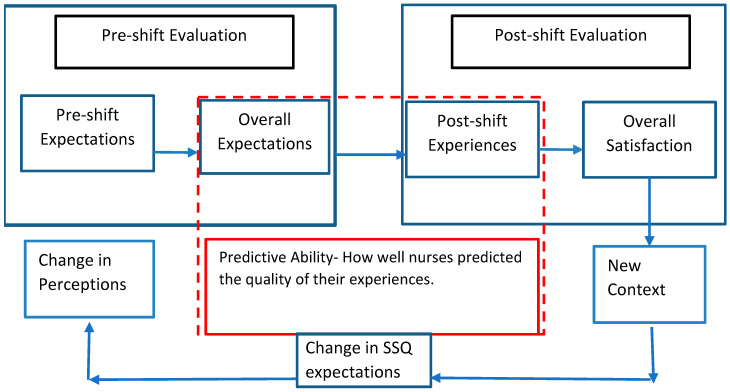
Prospective and retrospective relationships between the SDQ factors of nursing students.

Figure 1 illustrates the dynamic nature of the SSQ framework, showing how nurses’ perceptions—whether supported by actual experiences or not—influence their expectations and satisfaction. As students progress through each clinical shift, new experiences reshape their expectations for future shifts. These revised expectations inform how they interpret subsequent clinical contexts and determine the importance they assign to various stressors and supports.

In essence, SSQ operates as a feedback loop: post-shift experiences modify pre-shift expectations, which in turn affect how future shifts are perceived and managed. Positive or negative encounters can significantly alter students’ priorities and perceptions, leading to evolving strategies for coping, learning, and evaluating shift quality. This ongoing change in context also explains inconsistencies in predictive accuracy, and students’ prior experiences can bias how they anticipate the quality of upcoming shifts.

### 3.8. Ethical Considerations

This study received approval from the ethics committee of Auburn University and was conducted in accordance with the Declaration of Helsinki (institutional review board [IRB] protocol reference No. 20-092 EX 2206). On the first page of the survey, along with a consent form, the participants were informed of the purpose of this study and any potential risks. They were assured that no personally identifiable information would be collected. By completing the survey, they provided informed consent, and those who chose to proceed with the survey received USD 20 compensation for their time.

## 4. Results

### 4.1. Descriptive Statistics

Table 1 shows the participants’ descriptive statistics. The participants’ mean age was 21.86 years. Among the participants, 14% (*n* = 42) were male, and the remaining 86% (*n* = 262) were female. Regarding academic semester, 37% (*n* = 111) of the participants were in the fourth semester, 34% (*n* = 103) were in the second semester, and 29% (*n* = 90) were in the third semester. In terms of shift type, 35% (*n* = 105) were in the first shift, 30% (*n* = 94) were in the second shift, and 35% (*n* = 105) were in the third shift. Finally, regarding the participants’ clinical preceptors, 52% (*n* = 158) were part-time clinical faculty, while 48% (*n* = 146) were full-time faculty.

### 4.2. Correlation

Table 2 and Table 3 present the correlations between variables measured before and after the clinical shift, respectively. Before the shift (Table 2), unit type was positively associated with feeling comfortable with patient characteristics. Collegiality showed positive relationships with knowledge and skills, comfort with assigned tasks, patient characteristics, internal environment, organizational interaction with clinical faculty or preceptors, and the external environment. Knowledge and skills were positively associated with task comfort, patient characteristics, and faculty interaction, while negatively associated with overall shift perception. Patient characteristics were positively linked to internal and external environments, stress-coping strategies, and age. Organizational interaction was positively correlated with overall perception. The external environment showed strong associations with stress-coping and overall perception, while stress-coping was also positively linked to overall perception and negatively associated with age.

After the shift (Table 3), fewer variables showed statistically significant relationships at the *p* < 0.01 level. Unit type showed a negative correlation with knowledge/skills (*r* = −0.16). Collegiality positively correlated with overall perception (*r* = 0.19). Feeling comfortable with the tasks assigned showed a negative correlation with organizational interaction with the clinical training faculty/preceptor (*r* = −0.25). External environment also showed a negative correlation with the internal environment (*r* = −0.17). Finally, stress-coping strategies were positively associated with overall perception (*r* = 0.22).

### 4.3. Paired T Tests

Table 4 presents the results of the paired *t* tests performed to compare the variables before and after the clinical shift to identify the variables that showed statistically significant changes in students’ perceptions.

The results show that the students accurately predicted the patient characteristics (*t* = 0.87, *p* = 0.38) and external environment (*t* = 0.15, *p* = 0.81), as no significant differences were found between their pre- and post-shift ratings. By contrast, all the other variables demonstrated statistically significant differences at the *p* < 0.001 level, indicating a mismatch between the students’ expectations and actual experiences. The pre-shift mean scores for unit type, collegiality, and knowledge and skills increased after the shift. However, the students rated aspects such as the internal environment, organizational interaction with clinical faculty, feeling comfortable with assigned tasks, stress-coping strategies, and overall shift quality lower after the shift, which suggests that their initial expectations were more favorable than their actual experiences.

### 4.4. Unit Type

The perceived quality of the unit type increased from a prior mean of 2.73 to a post-mean of 3.17 (*t* = −4.32, *p* < 0.001).

### 4.5. Collegiality

Collegiality was rated more positively after the shift (*M* = 2.96) than anticipated before the shift (*M* = 2.49), with the difference being statistically significant (*t* = −9.82, *p* < 0.001). This suggests that the nurses underestimated the level of collegial support they experienced during their shifts. More specifically, the score for the item “Expecting cooperation/interactions between nurses’ colleagues who share responsibilities” increased from 2.60 pre-shift to 3.03 post-shift, indicating that the actual collaboration exceeded the initial expectations. The score for the item “Expecting interactions with the primary nurse assigned to the patient” followed a similar trend, increasing from 2.63 to 2.92, showing that teamwork with the primary nurse was stronger than anticipated. Finally, the score for the item “Shift quality resulting from the number of patients assigned” had the largest gap, increasing from 2.46 to 3.13, which suggests that the nurses expected a heavier or more difficult workload than they experienced.

Knowledge/skills. The score for knowledge/skills showed a notable increase from 2.39 to 3.00 (*t* = −6.12, *p* < 0.001).

Feeling comfortable with the tasks assigned. The students initially overestimated their comfort with the assigned tasks, as reflected by a significant decrease in their mean score from 3.69 before the shift to 3.00 after the shift (*t* = 19.35, *p* < 0.001), indicating that the tasks were more challenging than they anticipated.

Across several related items, the students consistently overestimated their comfort and anticipated ease with tasks before their clinical shift, as evidenced by the notable decreases in their mean scores post-shift. For the item “Expecting to have flexibility/opportunities for breaks in time between tasks during the shift,” the mean score decreased from 3.77 pre-shift to 2.84 post-shift, reflecting a more demanding and less flexible experience than expected. Similarly, when asked, “Based on the acuity level of assigned patients, are you expecting to have a manageable shift?”, the mean score decreased from 4.04 to 3.04, which suggests that the shift was more intense than predicted. The score for the item “Expected comfort level finishing the task(s) in the presence of the clinical instructor” also showed a strong decline from 4.07 to 2.78, indicating that the students felt significantly less confident in performing tasks with supervision than they had anticipated. Lastly, the score for the item “Having additional time to discuss the patient’s treatment plan with the clinical instructor” showed the largest gap, from 4.48 to 3.08, highlighting that time constraints were greater than expected.

Internal environment. The students rated the internal environment more favorably before the intervention (*M* = 3.57) than afterward (*M* = 2.94), with a statistically significant difference (*t* = 14.96, *p* < 0.001). This difference was reflected across several areas. For example, the rating for “Temperature in the unit” decreased from 4.59 to 3.18. Similarly, the item “Cleanliness of clinical spaces” Decreased from 3.66 to 2.79. The expectation of interruptions from visitors while completing the tasks/interventions was rated 4.16 beforehand and 3.11 afterward. Lastly, the students’ anticipation of interacting with new technology during the shift decreased from 4.21 to 2.94.

Organizational interaction with clinical training faculty/preceptors. The students’ perceptions of organizational interaction with clinical training faculty and preceptors significantly declined after the intervention, with mean ratings decreasing from 4.00 to 2.99 (*t* = 25.74, *p* < 0.001). Specifically, the ratings for “Expecting learning opportunities” decreased from 4.51 to 3.00, while those for “Expecting guidance level from the clinical instructor” decreased from 4.51 to 2.94. The students also reported a decrease in score for “Expected level of support by hospital staff” (from 4.16 to 3.15) and “Expected interaction with the primary nurse assigned to the patient” (from 4.57 to 2.84). In addition, perceptions regarding “Expecting staff shortage” declined slightly from 3.57 to 2.81.

Stress-coping strategies. The students’ use of stress-coping strategies significantly declined from a mean of 3.46 to 3.03 (*t* = 14.55, *p* < 0.001). This decrease was evident across multiple items. For instance, the ability to “adopt different strategies to solve problems” decreased from 4.47 to 3.16. Similarly, ratings for “Making plans, listing priorities, and solving stressful events” decreased from 4.00 to 2.85. The use of “past experiences to solve problems” also decreased, from 4.04 to 2.94. Finally, maintaining “an optimistic and positive attitude during the shift” showed a reduction from 3.61 to 3.00.

Overall prediction of shift quality. The overall perception of the experience decreased from 4.14 to 3.03 (*t* = 12.22, *p* < 0.001).

### 4.6. Linear Regression

In this section, three separate regression analyses were conducted to identify the most important variables that influenced students’ perceptions of shift quality:

1. The first model used pre-shift independent variables to predict pre-shift overall shift quality.
2. The second model used post-shift independent variables to predict post-shift overall shift quality.
3. The third model used pre-shift independent variables to predict post-shift overall shift quality to determine whether the students’ expectations could accurately predict their actual shift experience.

Table 5 presents a regression model in which all variables, including the dependent variable (“Overall expected shift quality”), were measured before the shift. The aim was to identify which aspects of the students’ expectations significantly influenced their perceived quality of their upcoming shift. The analysis revealed that the most important predictors were organizational interaction with the clinical faculty/preceptors, knowledge/skills, the external environment, and the internal environment (*p* < 0.05 for all). While organizational interaction and external environment showed a positive association with expected shift quality, knowledge/skills and internal environment were negatively associated.

**Figure 2:**
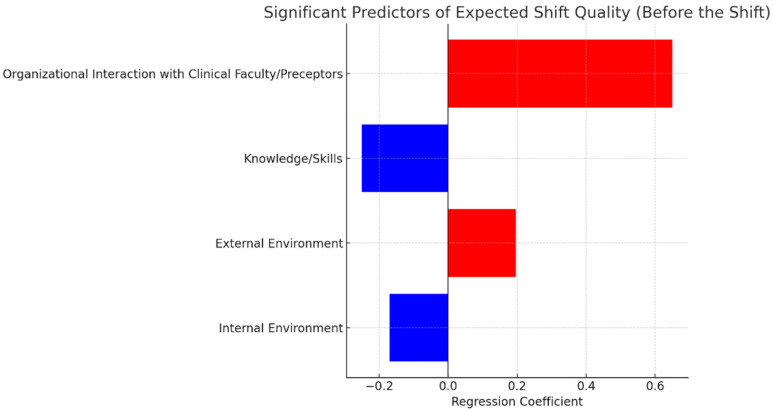
Most important predictors of expected overall shift quality based on pre-shift responses.

Figure 2 displays the significant predictors in ascending order of their regression coefficients. Positive predictors are shown in red bars on the right-hand side, while negative predictors appear in blue on the left-hand side. The most significant variable was “Organizational interaction with clinical faculty/preceptors,” followed by “External environment,” “Internal environment,” and “Knowledge/skills.”

Table 6 presents a regression model in which all variables, including the dependent variable (“Overall shift quality”), were measured after the shift. The objective was to examine which aspects of the students’ actual clinical experiences significantly influenced their evaluation of the shift. The analysis identified four statistically significant predictors (*p* < 0.01): stress-coping strategies, collegiality, patient characteristics, and unit type. Among these, stress-coping strategies and collegiality were positively associated with higher ratings of shift quality. By contrast, patient characteristics and unit type were negatively associated.

**Figure 3:**
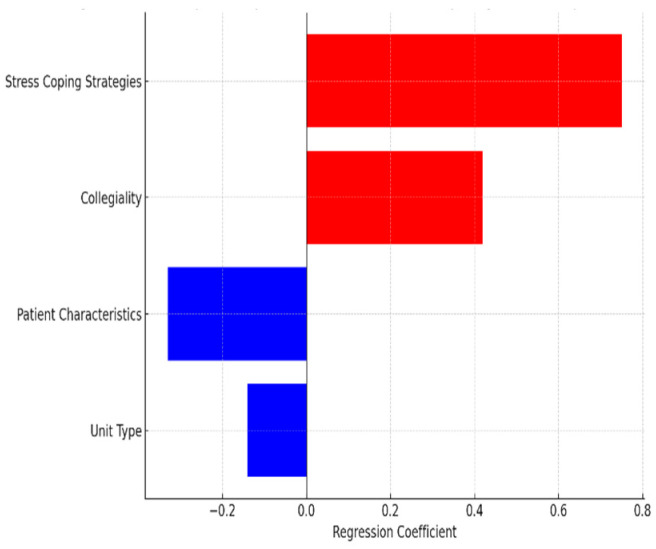
The most important predictors of overall shift quality based on post-shift responses.

On the basis of the results illustrated in Figure 3, the most important predictor of overall shift quality after the shift was stress-coping strategies, followed by collegiality, patient characteristics, and unit type.

A regression analysis was performed using the independent variables measured before the shift to predict the overall shift quality reported after the shift. The purpose of this analysis was to identify which aspects of the students’ pre-shift expectations could reliably predict their actual experiences throughout the day. However, the results showed that none of the pre-shift variables were statistically significant predictors of post-shift overall shift quality.

## 5. Discussion

This study investigated the predictive ability of nursing students to assess their own stressors and overall shift quality using a novel SSQ framework. This study investigated the relationship between nursing students’ pre-shift expectations and post-shift experiences during clinical training, revealing meaningful discrepancies between anticipated and actual experiences. The analysis highlights how students’ mental models evolve over the course of a single shift, particularly in response to organizational, interpersonal, and environmental factors. The analysis revealed both consistencies and discrepancies between the students’ pre- and post-shift perceptions across several critical domains. These findings offer meaningful insight into the psychological, environmental, and organizational complexities that shape clinical education in nursing.

### 5.1. Pre-Shift Perceptions: Structured and Interconnected Expectations

Prior to their clinical shifts, the nursing students demonstrated a highly structured and interconnected understanding of their work environment. This was evidenced by multiple statistically significant correlations across the key domains. In particular, collegiality emerged as a central driver of perceived shift quality, demonstrating strong associations with feeling comfortable with tasks (*r* = 0.40), perceptions of patient characteristics (*r* = 0.33), the internal environment (*r* = 0.17), organizational interaction (*r* = 0.22), and the external environment (*r* = 0.20). These results suggest that students viewed teamwork not as a discrete factor but as foundational to their entire clinical experience. This likely reflects a socially anchored coping orientation, wherein interpersonal support is relied upon more heavily than institutional structures or individual skills [34].

Knowledge and skills also positively correlated with feeling comfortable with tasks (*r* = 0.41) and organizational interaction (*r* = 0.35), reinforcing the role of self-efficacy in shaping clinical confidence [35]. Students who perceived themselves as more competent anticipated smoother task execution and more effective engagement with clinical faculty. These findings align with Bandura’s (1997) theory of self-efficacy, which posits that confidence in one’s capabilities influences how challenges are approached and managed [35].

Moreover, patient characteristics significantly correlated with both the external environment (*r* = 0.25) and coping strategies (*r* = 0.21), indicating that the students viewed more complex patient assignments as emotionally and physically demanding. This relationship suggests that students factored in their own emotional and physical readiness, such as fatigue, sleep quality, and stress levels, when anticipating the difficulty of patient care. Such perceptions underscore an early awareness of the emotional labor involved in nursing and the need for proactive coping mechanisms [36].

Before the shift, students approached their clinical assignments with structured expectations related to collegial support, task readiness, and environmental comfort. These expectations were likely influenced by classroom instruction, prior clinical exposure, and peer interactions. They represent students’ internalized models of what a successful shift should look like, emphasizing optimism and confidence in their ability to navigate stress and clinical demands. However, this confidence may have been shaped in relatively controlled environments, with limited exposure to the intensity of and variability in real-time clinical work. As such, students may have underestimated the complexity of patient care, team coordination, and situational pressures.

### 5.2. Post-Shift Realities: Fragmentation of Perceptions

After the shift, the cohesive network of pre-shift expectations became notably fragmented. Fewer statistically significant relationships were observed, suggesting a disconnect between anticipated and actual experiences. Coping strategies remained positively correlated with overall shift satisfaction (*r* = 0.22), highlighting the importance of resilience in adapting to the unpredictable demands of clinical work. Collegiality also maintained a modest but significant correlation with shift quality (*r* = 0.19), reinforcing the protective role of peer support.

However, some correlations shifted in unexpected directions. Unit type became negatively associated with knowledge and skills (*r* = −0.16), which suggests that certain clinical environments may have exposed gaps in student competence or challenged their self-confidence. In addition, a negative correlation between the internal and external environments (*r* = −0.17) may reflect how physical discomfort or environmental stressors interact with psychological strain, further complicating the students’ perceptions of control or preparedness during the shift.

These findings point to a disruption in the students’ mental models over the course of the shift. Initially holistic and optimistic, their perceptions became more fragmented and inconsistent under real-world pressures, indicating a need for stronger scaffolding in the transition from theoretical understanding to clinical execution [37].

### 5.3. Role of Organizational Interaction and Faculty Support

The regression analysis revealed that organizational interaction with clinical faculty was the strongest predictor of overall shift quality before the shift, whereas stress-coping strategies became the most significant predictor after the shift. The pre-shift emphasis on faculty interaction underscores the importance of structured mentorship and guidance in fostering student confidence. This aligns with the existing literature that identifies faculty accessibility and feedback as critical to clinical learning outcomes [26,38].

However, the post-shift decline in perceived support suggests that students often felt underserved or unsupported during the actual clinical experience. This may reflect either the limited availability of clinical faculty in high-pressure environments or a misalignment between student expectations and the realities of faculty engagement. Such discrepancies highlight the need for clearer communication of roles, responsibilities, and realistic support expectations in clinical education.

### 5.4. Coping Strategies and Psychological Flexibility

One particularly revealing outcome was the decline in the perceived effectiveness of stress-coping strategies following the shift. Although students began the day with a clear intention to manage stress through problem-solving, emotional regulation, and seeking peer support, the clinical realities they encountered—marked by unpredictability, emotional intensity, and time pressure—often undermined those plans. The demanding nature of the environment left little room for deliberate coping, causing even well-prepared students to feel overwhelmed. This observation echoes prior research suggesting that high-stress healthcare settings can diminish proactive coping due to fatigue, limited autonomy, and cognitive overload [4,39]. Importantly, coping strategies still proved to be strong predictors of post-shift satisfaction, underscoring their essential role in building resilience and promoting psychological well-being. These findings point to a dual imperative: equipping students with robust coping techniques and ensuring clinical environments support their use in real-time practice.

### 5.5. Implications for Training and Practice

The results of this study emphasize the need for nursing education programs to move beyond technical instruction and address the full spectrum of clinical demands, emotional, organizational, and environmental, that students must navigate. To better support students in real-world settings, we recommend the following:

Stress inoculation and simulation: Integrate stress inoculation training and high-fidelity simulation scenarios into clinical preparation. These allow students to practice coping strategies in safe, controlled environments that mimic the time pressure and unpredictability of actual clinical work [39].

Active and visible mentorship: Clarify faculty roles and increase visibility during clinical rotations to enhance perceived organizational support. Real-time mentorship—rather than sporadic check-ins—can bridge the gap between pre-shift expectations and in-shift realities [40].

Structured reflection and debriefing: Establish guided debriefing sessions and reflective journaling as part of the clinical routine. These practices enable students to recalibrate expectations, process their emotional responses, and internalize learning outcomes [41].

SSQ as a formative tool: Use the SSQ framework as a low-burden formative assessment to monitor student stress levels, satisfaction, and coping over time. Tracking this data can identify early signs of burnout or disengagement and inform continuous curriculum improvements [42].

Supportive clinical conditions: Ensure that students not only learn coping techniques but also have the time, autonomy, and psychological safety to apply them during their shifts. This requires collaboration between educational institutions and clinical sites to cultivate supportive work environments.

Interestingly, although students rated their knowledge and skills higher after the shift, this domain was negatively associated with overall satisfaction. This paradox may indicate that students who consider themselves highly competent may still face unexpected barriers that challenge their confidence. It also suggests that clinical competence alone is insufficient to mitigate dissatisfaction when environmental and emotional support are lacking. This underscores the complexity of nursing education and highlights the importance of balancing skill acquisition with resilience-building and adaptive capacity.

### 5.6. Limitations

This study has several limitations that should be acknowledged to ensure a balanced interpretation of the findings.

First, the data collection relied exclusively on nursing students’ self-reported perceptions and emotional assessments immediately before and after their clinical shift. Although this approach captures subjective experience, it introduces potential sources of bias such as recall bias, social desirability bias, and influences stemming from the teacher–student relationship. Students may, for instance, unconsciously align their post-shift responses with their expectations, or respond in ways they believe are professionally desirable.

Second, the dynamic nature of clinical environments introduces variability that is difficult to account for within the study design. Factors such as patient acuity, staffing ratios, unexpected clinical events, and institutional workflows fluctuate in real time and may influence students’ experiences beyond what can be measured in structured questionnaires.

Third, the use of linear regression models allows for the identification of associations between variables but does not establish causal relationships. For example, while coping strategies were found to be significant predictors of post-shift satisfaction, we cannot infer a direct causal pathway. Interpretations must therefore remain correlational.

Fourth, the sample was geographically and institutionally limited, reducing the generalizability of results. Students from other regions or programs might differ in terms of clinical preparation, support systems, or cultural expectations. Multi-site and cross-cultural studies are recommended to validate and expand upon these findings.

Fifth, although this study focused on immediate pre- and post-shift responses, it did not investigate how these experiences influence long-term learning, adaptation, or emotional resilience. Future research could benefit from longitudinal or mixed-method designs that trace development across multiple shifts or semesters.

Despite these limitations, this study offers several important contributions. It highlights the value of using short-cycle tools like the SSQ to assess student well-being and satisfaction in real time. It also provides actionable insights into how expectations and coping strategies impact students’ experiences during clinical training. Moreover, it suggests that certain factors such as the type of unit, familiarity with the clinical environment, use of user-friendly tools and technologies, and alignment with preferred medical teams can be proactively managed to support a more positive and confidence-building experience. Educational leaders may consider assigning students to clinical contexts where they feel psychologically and technically prepared, thereby enhancing not only satisfaction but also learning and resilience.

These findings reinforce the need for adaptive educational strategies that are responsive to the evolving realities of clinical work. By understanding the conditions that promote positive student experiences, nurse educators can design more supportive, efficient, and reflective clinical learning environments. Recognizing and addressing the gaps between pre-shift expectations and post-shift experiences can lead to more responsive and supportive educational strategies, ultimately producing more competent and resilient nursing professionals. That demands not only technical proficiency but also emotional preparedness, interpersonal effectiveness, and adaptive thinking. Recognizing and addressing the gaps between pre-shift expectations and post-shift experiences can lead to more responsive and supportive educational strategies, ultimately producing more competent and resilient nursing professionals.

## 6. Conclusions

This study evaluated nursing students’ ability to anticipate and assess the quality of their clinical shift experiences using a newly developed SSQ framework. The findings reveal a notable gap between students’ expectations and their actual shift experiences, particularly in areas such as faculty support, task readiness, and stress management. While peer collaboration and unit collegiality exceeded expectations, real-time clinical demands challenged students’ preparedness and coping capacity.

These insights contribute to the academic field by highlighting the dynamic nature of perceived stress and satisfaction within a single shift. The SSQ framework offers a practical tool to capture these fluctuations and can inform targeted educational interventions. Nursing programs may benefit from incorporating this framework to monitor student experiences in real time, support reflective practices, and better prepare students for the complexities of clinical environments.

By aligning educational strategies with the realities of clinical practice, this study underscores the importance of fostering emotional resilience, enhancing faculty engagement, and promoting team-based support. These efforts are essential to preparing competent, confident, and emotionally resilient future nurses.

## Figures and Tables

**Table 1:** Descriptive analysis of the participants.

Sociodemographic Variable	Number of Participants (*n* = 304), *n* (%)
Age (years), mean	21.86
Sex	
Male	42 (14)
Female	262 (86)
Semester	
Second	103 (34)
Third	90 (29)
Fourth	111 (37)
Shift Type	
First (7:00 PM to 3:00 AM)	105 (35)
Second (3:00 PM to 11:00 PM)	94 (30)
Third (11:00 PM to 7:00 AM)	105 (35)
Clinical Faculty	
Full-time	146 (48)
Part-time	158 (52)

**Table 2:** Correlation between the variables before the shift.

Variable	1	2	3	4	5	6	7	8	9	10	11
Unit type (1)	1	0.12 *	0.00	0.11	0.20 **	0.02	−0.14 *	0.03	0.13 *	−0.00	0.04
Collegiality (2)		1	0.19 **	0.40 **	0.33 **	0.17 **	0.22 **	0.20 **	0.14 *	0.04	0.12 *
Knowledge/skills (3)			1	0.41 **	0.22 **	0.09	0.35 **	0.11	0.01	−0.17 **	−0.04
Feeling comfortable with the tasks assigned (4)				1	0.37 **	0.23 **	0.38 **	0.15 **	0.14 *	0.10	0.00
Patient characteristics (5)					1	0.17 **	0.12 *	0.25 **	0.21 **	0.036	0.20 **
Internal environment (6)						1	0.30 *	0.08	0.14 *	0.00	0.03
Organizational interaction with clinical training faculty/preceptor (7)							1	0.00	0.05	0.21 **	0.14 *
External environment (8)								1	0.44 **	0.30 **	−0.07
Stress coping strategies (9)									1	0.16 **	−0.17 **
Overall (10)										1	−0.12 *
Age (11)											1

* *p* < 0.05, ** *p* < 0.01.

**Table 3:** Correlation between the variables after the shift.

Variable	1	2	3	4	5	6	7	8	9	10	11
Unit type (1)	1	0.02	−0.16 **	−0.06	−0.11	0.0	0.03	0.12 *	0.01	−0.12 *	−0.08
Collegiality (2)		1	−0.04	0.09	0.03	0.03	−0.07	0.09	0.11	0.19 **	−0.01
Knowledge/skills (3)			1	0.07	−0.10	−0.09	0.04	−0.09	0.04	0.00	−0.06
Feeling comfortable with the tasks assigned (4)				1	−0.08	−0.01	−0.25 **	−0.09	0.14 *	0.08	0.07
Patient characteristics (5)					1	0.02	−0.07	−0.05	0.03	−0.11	0.06
Internal environment (6)						1	−0.13 *	−0.17 **	−0.07	−0.11	−0.05
Organizational interaction with the clinical training faculty/preceptor (7)							1	0.02	−0.04	0.07	0.12
External environment (8)								1	0.08	0.02	0.03
Stress-coping strategies (9)									1	0.22 **	0.06
Overall (10)										1	0.04
Age (11)											1

* *p* < 0.05, ** *p* < 0.01.

**Table 4:** Paired T test between the pre- and post-shift variables.

Variable	T Statistics	Prior Mean	Post Mean	*p* Value
Unit type	−4.32	2.73	3.17	<0.001 *
Collegiality	−9.82	2.49	2.96	<0.001 *
Knowledge/skills	−6.12	2.39	3.00	<0.001 *
Feeling comfortable with the tasks assigned	19.35	3.69	3.00	<0.001 *
Patient characteristics	0.87	3.20	3.15	0.38
Internal environment	14.96	3.57	2.94	<0.001 *
Organizational interaction with the clinical training faculty/preceptors	25.74	4.00	2.99	<0.001 *
External environment	0.1538	2.97	2.96	0.81
Stress-coping strategies	14.55	3.46	3.03	<0.001 *
Overall	12.22	4.14	3.03	<0.001 *

* *p* < 0.01.

**Table 5:** Regression model before the shift.

Variable	Coef	STD Err	T	$p\;>\;\left|t\right|$	[0.025]	[0.975]
Constant	1.47	0.60	2.48	0.014	0.305	2.65 *
Unit type	0.02	0.04	0.42	0.67	−0.06	0.09
Collegiality	−0.078	0.08	−0.96	0.34	−0.24	0.08
Knowledge/skills	−0.25	0.04	−6.14	0.00	−0.34	−0.17 *
Feeling comfortable with the tasks assigned	0.22	0.11	1.93	0.05	−0.00	0.45
Patient characteristics	−0.02	0.061	−0.39	0.70	−0.14	0.098
Internal environment	−0.17	0.08	−2.027	0.04	−0.32	−0.01 *
Organizational interaction with clinical training faculty/preceptors	0.65	0.115	5.69	0.00	0.43	0.88 *
External environment	0.19	0.03	5.89	0.00	0.13	0.26 *
Stress-coping strategies	0.09	0.13	0.14	0.88	−0.24	0.28

* Significant variables, *p* < 0.01.

**Table 6:** Regression model after the shift.

Variable	Coef	STD Err	T	$p\;>\;\left|t\right|$	[0.025]	[0.975]
Constant	0.83	1.36	0.61	0.54	−1.84	3.51
Unit type	−0.14	0.05	−2.60	0.01	−0.25	−0.03 *
Collegiality	0.42	0.13	3.29	0.01	0.17	0.68 *
Knowledge/skills	−0.05	0.05	−0.88	0.38	−0.16	0.06
Feeling comfortable with the tasks assigned	0.14	0.19	0.76	0.44	−0.22	0.50
Patient characteristics	−0.33	0.4	−2.28	0.02	−0.61	−0.05 *
Internal environment	−0.25	0.15	−1.62	0.10	−0.55	0.05
Organizational interaction with the clinical training faculty/preceptors	0.23	0.14	1.62	0.10	−0.05	0.51
External environment	−0.03	0.01	−0.28	0.77	−0.22	0.16
Stress-coping strategies	0.75	0.21	3.54	0.00	0.33	1.17 *

* Significant variables, *p* < 0.01.

## Data Availability

The data supporting this study is not publicly available due to privacy restrictions.
